# Synergism of Feeding and Digestion Regulated by the Neuropeptide F System in *Ostrinia furnacalis* Larvae

**DOI:** 10.3390/cells12010194

**Published:** 2023-01-03

**Authors:** Jiajia Zhao, Yu Song, Xuemin Jiang, Lei He, Liya Wei, Zhangwu Zhao

**Affiliations:** 1 Department of Entomology, MOA Key Lab of Pest Monitoring and Green Management, College of Plant Protection, China Agricultural University, Beijing 100193, China; 2 College of Life Sciences, Hebei University, Baoding 071002, China

**Keywords:** NPF, insulin pathway, feeding, digestion, *O. furnacalis*

## Abstract

Feeding is crucial for the growth and survival of animals, including humans, but relatively little is known about how it is regulated. Here, we show that larval feeding in *Ostrinia furnacalis* is regulated by neuropeptide F (NPF, the homologous peptide of mammalian NPY) via the insulin signalling pathway in the midgut. Furthermore, the genes *pi3k* and *mtor* in the insulin pathway positively regulate α-amylase and lipase of the midgut by recruiting the transcription factors *c-Myc* and *PPARγ* for binding to the promotors of these two enzymes. Importantly, we find that the feeding behaviour and the digestive system of midgut in *O. furnacalis* larvae are closely related and interactive in that knocking down α-amylase or lipase induces a reduction in larval feeding, while food-deprived larvae lead to fewer expressions of *α-amylase* and *lipase*. Importantly, it is the gut NPF that regulates the α-amylase and lipase, while variations of α-amylase and lipase may feed back to the brain NPF. This current study reveals a molecular feedback mechanism between feeding behaviour and the digestive system that is regulated by the conserved NPF via insulin signalling systems in the midgut of *O. furnacalis* larvae.

## 1. Introduction

Feeding is a very important behaviour which all animals (including humans) depend on for survival. Natural selection strongly acts on the feeding behaviour of animals [1], which is modifiable based on physiological need [2]. The regulation of feeding behaviours is highly conserved between insects (such as *Ostrinia* and *Drosophila*) and mammals, and food-deprived animals urgently require food acquisition to maintain normal energy consumption.

Neuropeptides in animals play a central role in the regulation of many physiological processes, such as development, reproduction, and feeding [2,3,4]. Previous reports showed that some neuropeptides seriously impact the growth and development of insects by regulating metabolism and physiological functions, such as adipokinetic hormone (AKH), allatostatin (AT), allatotropin (AST), and prothoracicotropic hormone (PTTH) [5]. However, insects have a class of NPY-like neuropeptides (called neuropeptide F, NPF) which are homologous to the peptides of the mammalian NPY family and act on feeding through NPFR, a G-protein-coupled receptor. NPF was first identified in *Helicoverpa zea* [6], followed by *Drosophila melanogaster* [7], *Schistocerca grearia* [8], *Aedes aegypti* [9], *Anopheles gambiae* [10], *Locusta migratoria* [11], *Bombyx mori* [12], *Reticulitermes flavipes* [13], and *Helicoverpa assulta* [14]. One important function regulated by NPF is feeding behaviour, which has been reported in invertebrates [15] and in many insects, such as *D. melanogaster* [16], *Heliothis assulta* [14], *Helicoverpa armigera* [17], *Acyrthosiphon pisum* [18], *L. migratoria* [19], *Grapholita molesta* [20], *Ostrinia furnacalis* [21,22,23,24], etc. We previously reported that NPF is mainly expressed in the larval midgut and brain in *H. assulta* [14], *H. armigera* [17], and *O. furnacalis* [21,22].

The insulin-like signalling pathway in insects acts as a sensor of nutritional status that can affect digestion, feeding, development, reproduction, and diverse anabolic processes [25,26,27]. *D. melanogaster* encodes seven insulin-like proteins but appears to have a single insulin-like receptor [2]. In *D. melanogaster*, the NPF system has been found to increase feeding through the insulin-like receptor/PI3K/S6K pathway in the fat body [28], in which rapamycin (mTOR) is a downstream target of PI3K that controls cellular protein synthesis and energy homeostasis [29]. However, relatively little is known about the inherent regulatory mechanism of feeding behaviour.

α-amylase is an important digestive enzyme in the growth and development of insects, and its main function is to participate in the hydrolysis of carbohydrates [30,31,32]. In insects, only α-amylase has been found to hydrolyse glycogen and starch [33,34], and α-amylase activity has been described in several insect orders, including Coleoptera, Hymenoptera, Diptera, Lepidoptera and Hemiptera [35,36,37,38,39]. Lipase is a widely used catalyst that can gradually hydrolyse lipids into fatty acids [40,41,42]. A correlation between food intake and enzyme activity has been reported in *A. aegypti* [43] and *Leucophaea* [44] in which feeding stimulates lipase secretion, whereas starvation reduces lipase secretion in the cricket *Gryllus bimaculatus* [45]. However, the interaction between feeding and digestion functions is still unknown.

In previous reports, we showed the details of how the feeding behaviour of *O. furnacalis*, an important agricultural pest, is regulated by NPF in that NPF mainly expresses in the midgut and brain, and the brain NPF regulates larval feeding via JH signal pathway and the sensitivities of the sensilla styloconica in the mouthparts [14,17,21,22,23,24]. In this study, we further focused on midgut signal pathway involved in the feeding regulation of *O. furnacalis*, which would be important for clarifying the complex molecular mechanism in feeding regulation.

## 2. Materials and Methods

### 2.1. Insect Rearing

The eggs of *O. furnacalis* were obtained from the laboratory of Dr. Zhenying Wang (Chinese Academy of Agricultural Sciences, Beijing, China). After hatching, the larvae were reared according to methods previously described [21]. All stages of *O. furnacalis* were kept at 28 ± 1 °C and 60% relative humidity under a photoperiod of 16 L:8 D.

### 2.2. Drosophila Strains and Rearing

All *Drosophila* stocks were reared on standard cornmeal/agar *Drosophila* food at a temperature of 25 °C and 60% relative humidity under a photoperiod of 12 L:12 D. UAS-α-amylase-RNAi (CG18730), UAS-lipase-RNAi (CG8823), and UAS-NPF-RNAi (THU2569) were purchased from the fruit fly centre of Tsinghua University, MyoIA-Gal4 was obtained from the laboratory of Yi Rao (Peking University, Beijing, China), UAS-NPFR-RNAi was obtained from the laboratory of Yan Zhu (Chinese Academy of Sciences, China), and nsyb-Gal4 (51941) was purchased from Bloomington Drosophila Stock Center.

### 2.3. RNA Extraction and cDNA Synthesis

Total RNA was extracted from 5th instar larvae using RNAiso Plus (TaKaRa, Dalian, China). The concentration and integrity of RNA were detected using a Nanodrop2000c spectrophotometer (Thermo Fisher Scientific, West Palm Beach, FL, USA) and 1% agarose gel electrophoresis, respectively. Reverse transcription was performed with 1 µg of total RNA using the PrimeScriptTM RT Reagent Kit with gDNA Eraser (Perfect Real Time) (TaKaRa, Dalian, China). The synthesized first-strand cDNA was stored at −20 °C until use.

### 2.4. Synthesis of Double-Stranded RNA for Artificial Diet Treatment

To produce a large batch of ds*RNA* for targeted gene silencing, efficient ds*RNA* production was constructed via engineered bacteria according to a previously described procedure [46] in which the sequences of *npf* (GenBank accession no: XM_028311349.1), *npfr* (GenBank accession no: XM_028321656.1), *pi3k* (XM_028322865.1) and *mtor* (XM_028311795.1) in *O. furnacalis* were obtained from NCBI (https://www.ncbi.nlm.nih.gov/) (accessed on 12 March 2019). To construct the expression vectors, specific primers with restriction sites (ds*NPFR*/*PI3K*/*mTOR*-sequence 1, EcoRI and XbaI; ds*NPFR*/*PI3K*-sequence 2, XhoI and XbaI; and ds*mTOR*-sequence 2, HindIII and XbaI) (Supplementary Table S1) were used to amplify sequence 1 and sequence 2 of the target genes from cDNA. PCR were conducted with the following program: 94 °C for 3 min; followed by 30 cycles of 94 °C for 30 s, 55 °C for 30 s and 72 °C for 1 min; with a final extension at 72 °C for 10 min. Purified PCR products were ligated into the *pEASY*-Blunt Zero cloning vector (TransGen Biotech, Beijing, China), and positive clones were sequenced. Sequence 1 and 2 constructs were digested with corresponding restriction endonucleases at 37 °C for 2 h and separated on the gel. The expected band was purified, and two reverse complementary sequences of the target gene were ligated into the linearized expression vector pET28a (+) with T4 DNA ligase (NEB, Ipswich, MA, USA), and sequence 1 contained a loop structure; pET28a (+) was gifted by Dr. Jie Shen (China Agricultural University). The ligation products were transformed into DH5α (TransGen Biotech, Beijing, China) to verify the insert sequences. The plasmids were then transformed into BL21 (DE3) RNase III- and incubated at 37 °C overnight. Single colonies were selected and cultured in 5 mL of Luria–Bertani (LB) liquid media supplemented with 50 mg/L kanamycin (Kan) and incubated at 37 °C overnight with shaking at 220 r/min. The 2.67 mL culture liquid was diluted into 400 mL LB liquid media supplemented with Kan followed by shaking at 37 °C, and the cells were grown until the optical density at 600 nm (OD600) reached 0.4. Isopropyl β-D-1-thiogalactopyranoside (IPTG) was then added to 1 mM and continuously incubated for another 5 h at 37 °C.

A total of 400 mL of cell culture was heated to 80 °C for 20 min, cooled to room temperature, and then centrifuged at 6000× *g* at 4 °C for 10 min. The bacterial pellet was resuspended in 15 mL of 75% absolute ethanol prepared with 10X phosphate buffer saline (10X PBS), shaken and then incubated for 5 min and centrifuged again at 6000× *g* at 4 °C for 10 min. The bacterial pellet was resuspended in 10 mL of 100 mM NaCl, shaken, incubated at 4 °C for 2 h, and centrifuged at 8000× *g* at 4 °C for 10 min to obtain dsRNA. The concentration and integrity of dsRNA were detected by a NanoDrop^®^ND-1000 spectrophotometer (Thermo Fisher Scientific, Inc. Waltham, MA, USA) and 1% agarose gel electrophoresis, respectively. Newly moulted 5th instar larvae were fed an artificial diet containing 0.02% ds*RNA* (20 mg ds*RNA* in 100 g diet).

### 2.5. Synthesis of Double-Stranded RNA for Injection Treatment

The prepared cDNAs were used as templates for the amplification of *α-amylase* and *lipase* genes. The *pEASY*-Blunt Zero cloning vector (TransGen Biotech, Beijing, China) was used for the gene cloning. The plasmids were extracted using a PurePlasmid Mini Kit (CWBio, Beijing, China). The dsRNAs of *α-amylase* and *lipase* were synthesized from the extracted plasmids with T7 promoter sequences using the T7 RiboMAX™ Express RNAi System (Promega, Madison, WI, USA) according to the manufacturer’s protocol. The *α-amylase* and *lipase* primers are listed in Supplementary Table S2. A total of 600 ng of ds*RNA* was injected into the lateral intersegmental membrane of 5th instar larvae between the third and fourth abdominal segments.

### 2.6. Quantitative Real-Time PCR

Larvae with different genes were separately knocked down using different dsRNA methods, in which total RNA from each dsRNA sample was isolated, and cDNA was synthesized from 1.0 µg of total RNA as described above. Three replicates were conducted for each sample.

qPCR was carried out using SuperReal PreMix Plus (SYBR Green) (Tiangen, Beijing, China) on the StepOne Real-Time PCR system (Applied Biosystem, Foster, CA, USA). All primers used are listed in Supplementary Table S3. The reference gene *actin* (GenBank number: XM_028306632.1) was used as the experimental control. qPCRs were performed in 20 μL volumes, which consisted of 10 μL of 2× SuperReal PreMix Plus (with SYBR Green I), 2 μL of 50× ROX Reference Dye, 1 μL of cDNA, 0.6 μL of each primer (10 μmol/L), and 5.8 μL of RNase-free H_2_O. For the products *OfurPI3K* and *OfurmTOR*, an initial denaturation at 95 °C for 10 min was first employed, followed by 40 cycles at 95 °C for 15 s, 60 °C for 30 s, and 72 °C for 30 s. For the products of other genes, 95 °C for 10 min was first applied followed by 40 cycles at 95 °C for 15 s and 60 °C for 1 min. The amplification efficiencies for *npf*, *npfr*, *pi3k*, *mtor*, *pdk*, *akt,* a*PKC*, *α-amylase*, *lipase*, *actin* in *O. furnacalis*, as well as *npf*, *α-amylase*, *lipase*, *actin* in *D. melanogaster* were 95.431%, 96.219%, 97.243%, 99.367%, 96.366%, 95.029%, 97.812%, 96.713%, 95.01%, 99.276%, 100.673%, 96.568%, 96.498%, and 97.952%, respectively. The melt curve of all genes was unimodal. The relative expression was calculated with the 2^−∆∆CT^ method [47]. Data are shown as the mean ± SE (standard error). A Student’s t test (Prism GraphPad) was used to compare the differences between genotypes.

### 2.7. Western Blot

To determine the expression effects of NPF protein levels, the coding sequences of *npf* were inserted into the linearized pET28a (+) expression vector. The construct containing *npf* genes was transformed into *E. coli* BL21 cells (Tiangen, Beijing, China) and induced with a final concentration of 1 mM IPTG for expression. The expression product was transferred to ABclonal Company to synthesize rabbit anti-NPF (ABclonal Technology, Wuhan, China). The midgut was homogenized and lysed in RIPA lysis buffer (CWBio, Beijing, China) supplemented with protease inhibitor cocktail. Protein contents were determined with a BCA Protein Assay Kit (Beijing BioDee Biotechnology, Beijing, China). Next, 20 μg of protein was separated with 15% SDS-PAGE and transferred to an Immobilon-P PVDF transfer membrane (Millipore, Bedford, MA, USA). After blocking with 5% skim milk in TBST at room temperature for 2 h, the membranes were incubated overnight at 4 °C with primary antibody specific to rabbit anti-NPF (1:500) and mouse anti-β-tubulin (1:2000). The membranes were washed in TBST and then incubated with HRP goat anti-rabbit IgG (H+L) and HRP goat anti-mouse IgG (H+L) (ABclonal Technology, Wuhan, China) (1:2000) at room temperature for 2 h. The signals were captured using the Azure Biosystems C600 (AZURE Biosystems, Dublin, CA, USA) and quantified using ImageJ (NIH, Bethesda, MA, USA).

### 2.8. Determination of Midgut Enzyme Activities

To test the activities of midgut digestive enzymes, crude enzyme liquids from larvae of different treatments were separately prepared by homogenizing and centrifuging 0.1 g of midgut sample with 1 mL of extracting solution (a kit from the Suzhou Comin Biotechnology, Suzhou, China). The activities of α-amylase (XM_028303102.1) and lipase (XM_028311845.1) were determined by the micromethod as described from the Assay Kits of α-amylase and lipase, respectively (Suzhou Comin Biotechnology, Suzhou, China). A BCA Protein Assay Kit (Beijing BioDee Biotechnology, Beijing, China) was selected as the standard.

### 2.9. Digestion and Feeding Assays

For reducing sugar and fatty acid assays, 0.1 g midgut samples from larvae of different treatments were separately collected and homogenized with 1 mL extracting solution. The assays were performed with kits according to the assay kits of reducing sugar and fatty acid, respectively (Suzhou Comin Biotechnology, Suzhou, China).

Food intake was assayed using methods from Edgecomb with slight modification [48]. To ascertain whether *npf*, *npfr*, *pi3k,* and *mtor* affect larval feeding behaviour, 5th instar larvae were fed an artificial diet containing 0.02% dsRNA. After 24 h, the larvae were switched to 100 g food containing 2.5% brilliant blue for 15 min (the absorption wavelength of brilliant blue is 625 nm), and then the larval midguts were homogenized in PBS buffer and centrifuged (13,000 rpm) for 10 min. The supernatants were transferred to a new tube and filtered using 0.22 μM syringe filters. Absorbance was measured at 625 nm. Each treatment was repeated at least three times with a minimum of 10 individuals per replicate.

### 2.10. Starvation Treatment of Larvae

Newly moulted 5th instar larvae were fed a normal artificial diet for 3 h and then transferred to 1% agar as a water source for 6 h of fasting. The normal feeding group was continuously fed with the artificial diet as previously described [Yue et al., 2017]. The larval midguts were sampled to detect the expression and enzyme activities of α-amylase and lipase. Each treatment was repeated at least three times with a minimum of 10 individuals per replicate.

### 2.11. Rescue Experiments

The PI3K inhibitor (LY294002) (2 µM) and activator (740 Y-P) (1 µM) (MedChemExpress, Monmouth Junction, NJ) were used to target the PI3K/mTOR pathway. Larvae were fed diets with LY294002 or 740 Y-P for 24 h, and ds*NPFR* containing activator 740 Y-P was used as the rescue treatment. Larval midguts were dissected and frozen after feeding for 24 h and were used for further analysis of *mtor* transcription, the enzyme activities of α-amylase and lipase, and the levels of reducing sugars and fatty acids.

### 2.12. Dual Luciferase Reporter Assay

The sequences corresponding to the *c-Myc* (XP_028175965.1) (amino acids 1-87) and *PPARγ* (XP_028158532.1) (amino acids 1–327) coding regions were subcloned into the pAc5.1b/V5/His expression vectors (Thermo Fisher Scientific, West Palm Beach, FL, USA). The promoter regions of *α-amylase* (−1914 to +86) and *lipase* (−1923 to +175) and their mutants were subcloned into pGL3 luciferase reporter vectors (Promega). The sequences of transcription factors in the promoter regions of *α-amylase* and *lipase* were mutated, which was achieved by the corresponding mutant primers with the promoter sequence as a template. Subsequently, 100 ng of pGL3-*α-amylase*, pAc5.1b- *c-Myc*, pGL3-*lipase*, and pAc5.1b- *PPARγ* was separately transfected into *Spodoptera frugiperda* cells (Sf9, from the laboratory of Shen Jie) and were kept at 25 °C in Sf-900™IIISFM (Thermo Fisher Scientific, West Palm Beach, FL, USA) supplemented with 10% foetal bovine serum (TIANHANG, Zhejiang, China). Cell transfections were carried out using X-tremeGENE HP DNA Transfection Reagent (Roche, Basel, Switzerland). After 48 h treatment, cells were rinsed twice with PBS (pH 7.4) and lysed with 1 X passive lysis buffer as supplied in the Dual-Luciferase^®^ Reporter Assay System (Promega), and then the Luciferase and Renilla luciferase activities were separately measured using a fluorescence spectrometer (Infinite F200Pro, Tecan, Switzerland) following the manufacturer’s instructions for the Dual-Luciferase^®^ Reporter Assay System (Promega). The sequences of the primers are shown in Supplementary Table S4.

## 3. Results

### 3.1. Construction and Expression of dsRNA Vectors

To produce large amounts of ds*RNA* for the study of feeding mechanisms, we constructed a ds*RNA* expression system according to the methods of Ma [46]. The amplified sequences of *npfr*, *pi3k*, and *mtor* were separately cloned and ligated into the *pEASY*-Blunt Zero cloning vector. Next, sequence 1 and sequence 2 of the corresponding genes were separately digested and ligated into pET28a (+). The constructed vector pET28-*npfr*/*pi3k*/*mtor*/*gfp* was transformed into BL21 (DE3) RNase III- to produce the target ds*RNA* (Supplementary Figure S1). T7-promoter-mediated RNA transcription was activated to transcribe a single-stranded RNA with two reverse complementary fragments that formed ds*NPF* (322 bp), ds*NPFR* (472 bp), ds*PI3K* (465 bp), ds*mTOR* (466 bp), and ds*GFP* (391 bp). The interference efficiencies of ds*NPF* on both mRNA and protein levels were verified using qPCR and western blot, respectively, in which levels of both the *npf* gene and NPF protein were significantly decreased (58%, *p* < 0.0001; and 30%, *p* < 0.01, respectively) in the ds*NPF*-treated larvae compared with those of the ds*GFP* larvae (Supplementary Figure S2a,b). The interference efficiencies of ds*NPFR*, ds*PI3K* and ds*mTOR* were also separately verified, all of which were significantly knocked down compared with those of the ds*GFP* larvae (Supplementary Figure S2c–e).

### 3.2. The Insulin Pathway in the Midgut Is Involved in NPF/NPFR-Mediated Feeding Regulation

**Figure 1:**
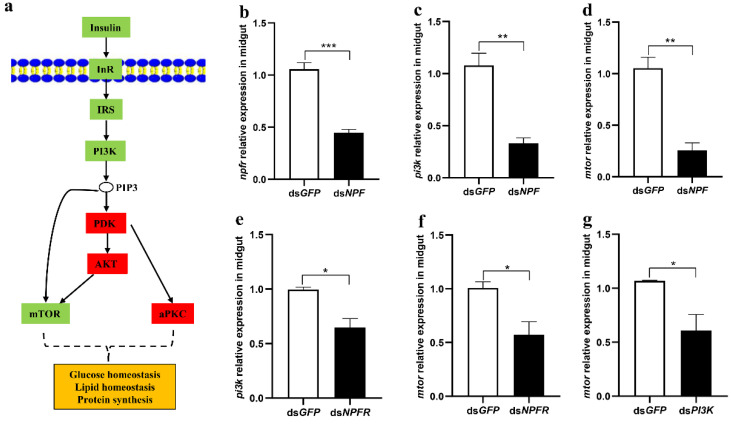
The relationship between *npf* and key genes in the insulin pathway in the midgut of *O. furnacalis*. (**a**) Diagram of insulin signalling pathways. (**b**–**g**) Transcript levels of *npfr*, *pi3k* and *mtor* in the midgut of 5th instar larvae with knockdown of NPF, NPFR, and PI3K, respectively, for 24 h. Each treatment was repeated 3 times with 10 individuals per replicate. Bars represent the mean ± SE. * *p* < 0.05, ** *p* < 0.01, *** *p* < 0.001.

Insulin is closely related to the growth and development of animals. To explore whether NPF regulation of larval feeding is related to the insulin signalling pathway, we measured the key genes in the insulin pathway after knocking down NPF, as shown in Figure 1a. The results showed that the levels of *npfr*, *pi3k,* and *mtor* in the midgut were significantly decreased by 58% (*p* < 0.001), 69% (*p* < 0.01), and 76% (*p* < 0.01), respectively, after NPF was knocked down for 24 h (Figure 1b–d). However, the *pdk*, *akt,* and *aPKC* levels were not impacted by ds*NPF*, increased by 29% and 12% and decreased by 1%, respectively (Supplementary Figure S3), indicating that the NPF system regulates larval feeding through PI3K directly to mTOR in the insulin pathway (Figure 1a, green colour) and not through the PI3K/PDK/AKT/mTOR or PI3K/PDK/aPKC pathways (Figure 1a, red colour).

Furthermore, we separately downregulated the expression of *npfr*, *pi3k,* and *mtor* and detected their downstream genes. The results showed that expressions of *npfr, pi3k, and mtor* in the insulin pathway were significantly reduced when larvae were knocked down with NPFR, and expressions of *pi3k* and *mtor* were significantly reduced when larvae were knocked down with PI3K (Figure 1e–g).

**Figure 2:**
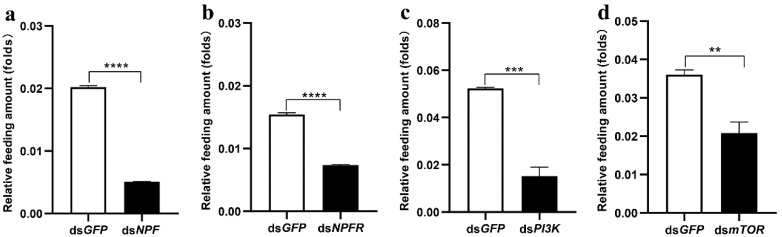
Feeding regulation by NPF, NPFR, PI3K, and mTOR. Relative feeding amount of 5th instar larvae with knockdown of NPF (**a**), NPFR (**b**), PI3K (**c**), and mTOR (**d**), respectively, for 24 h. Each treatment was repeated 3 times with 10 individuals per replicate. Bars represent the mean ± SE. ** *p* < 0.01, *** *p* < 0.001, **** *p* < 0.0001.

When larvae were downregulated with NPF, NPFR, PI3K, and mTOR, respectively, larval feeding was significantly decreased in all ds*RNA*-treated larvae compared to those of the ds*GFP* larvae (Figure 2) These data demonstrate that the insulin signalling pathway in the midgut serves as a downstream target of NPF/NPFR in feeding regulation.

### 3.3. NPF/NPFR Regulates Digestive Enzymes via the Insulin Pathway

**Figure 3:**
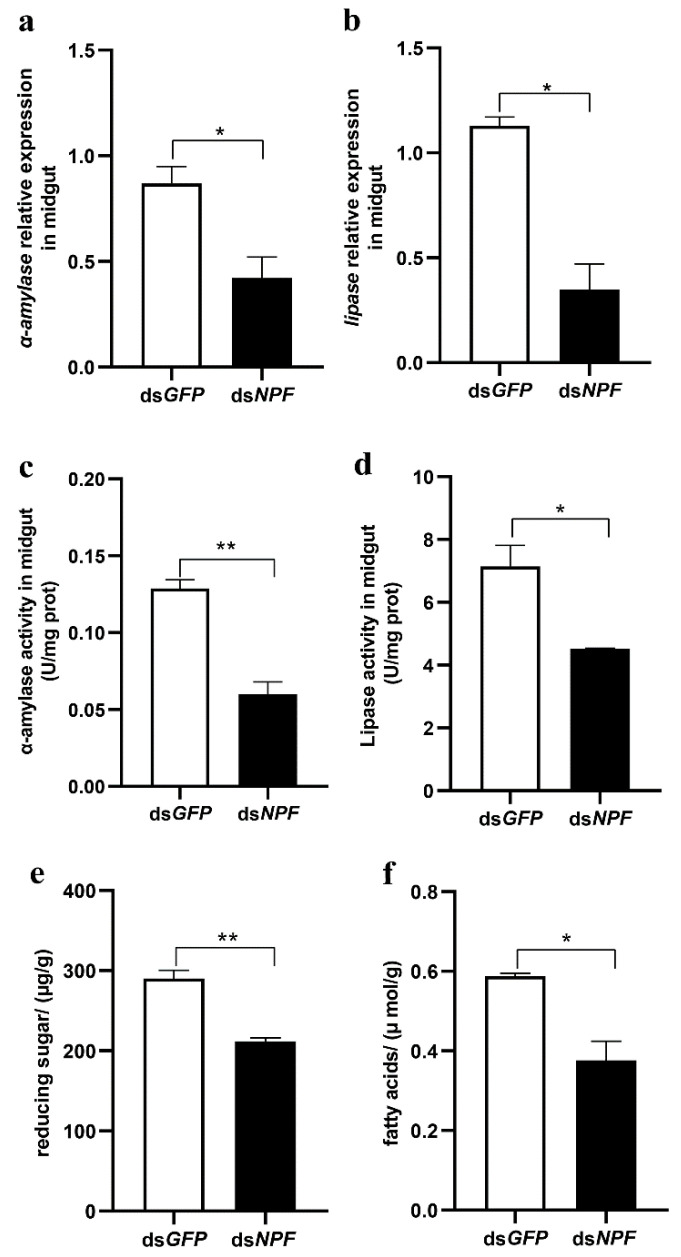
Roles of NPF with regard to larval digestion enzymes. (**a**–**d**) The expressions and enzyme activities of midgut α-amylase and lipase, respectively, in 5th instar larvae with NPF and GFP knockdown for 24 h. (**e**,**f**) The content of reduced sugars and fatty acids in the midgut of 5th instar larvae with NPF and GFP knockdown for 24 h. Each treatment was repeated 3 times with a minimum of 10 individuals each repeat. Bars represent the mean ± SE, * *p* < 0.05, ** *p* < 0.01.

To determine whether NPF is related to the digestive system, because the main role of the midgut is food digestion, we explored both the mRNA expression and activities of α-amylase and lipase by knocking down NPF via ds*RNA*. The results showed that both the expression and activity of α-amylase and lipase were significantly reduced (Figure 3a–d), and the contents of reducing sugars and fatty acids were significantly lower in the downregulated larvae (Figure 3e,f), indicating that these digestive enzymes are regulated by NPF in the midgut.

**Figure 4:**
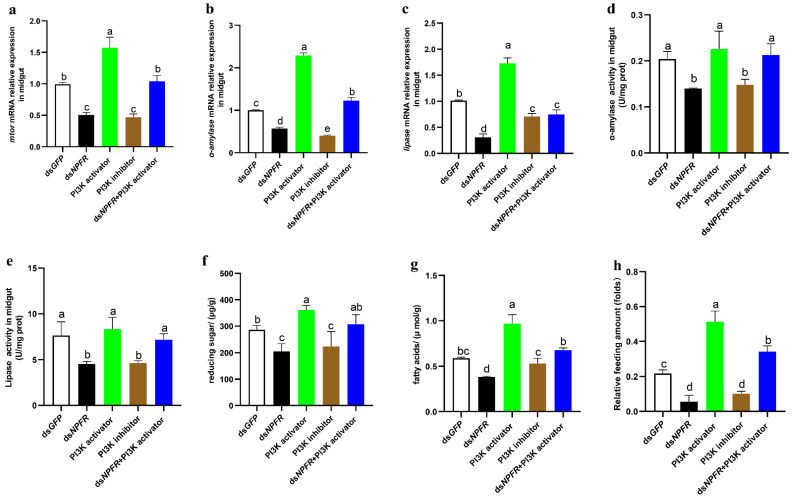
NPF/NPFR regulates digestion enzymes via the PI3K/mTOR pathway in the midgut. All treated 5th instar larvae were reared with the diets ds*NPFR*, PI3K activator 740 Y-P (1 µM), PI3K inhibitor LY294002 (2 µM), or ds*NPFR*+PI3K activator for 24 h. (**a**–**c**) Relative expression levels of *mtor*, α-amylase, and lipase in the midgut of treated larvae. (**d**,**e**) Enzyme activities of α-amylase and lipase in the midgut of treated larvae. (**f**,**g**) Contents of reduced sugars and fatty acids in the midgut of treated larvae. (**h**) Relative feeding amount in the midgut of treated larvae. Each treatment was repeated 3 times with a minimum of 10 individuals each repeat. Different letters above the bars indicate significant differences at *p* < 0.05 as determined by Duncan’s multiple range test.

To further ascertain whether NPF regulates the digestive system via the insulin pathway, we performed rescue experiments with an activator (740 Y-P) and an inhibitor (LY294002) of PI3K. The “740 Y-P” is a potent and cell-permeable PI3K activator, which readily binds GST fusion proteins containing both the N- and C-terminal SH2 domains of p85 but fails to bind GST alone. The “LY294002” is a broad-spectrum inhibitor of PI3K. The results showed that ds*NPFR* or 2 µM of PI3K inhibitors effectively knock down the expression of *mtor*, which is rescued by 1 µM of PI3K activator or ds*NPFR*+pi3k activator (Figure 4a). Furthermore, the expressions and activities of α-amylase and lipase, the contents of reducing sugars and fatty acids, and feeding were significantly inhibited by ds*NPFR* and PI3K inhibitor, which might be effectively rescued by PI3K activator and ds*NPFR+* PI3K activator (Figure 4b–h), respectively. These data demonstrate that the NPF-mediated insulin signalling pathway regulates larval feeding by controlling the digestive system in terms of both the expressions and activities of α-amylase and lipase.

### 3.4. Midgut Digestive Enzymes Are Closely Related to Larval Feeding

**Figure 5:**
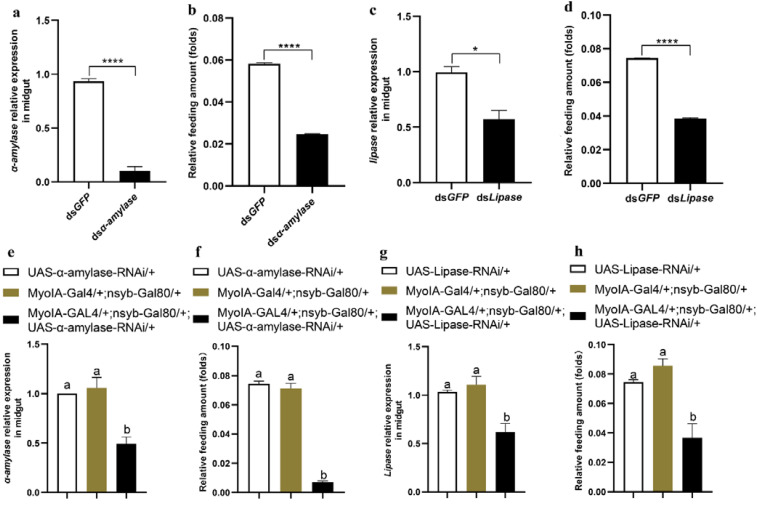
Effects of α-amylase and lipase on larval feeding. (**a**,**c**) Relative expression levels of α-amylase and lipase in the midgut after knockdown of α-amylase or lipase, respectively, for 3 h in 5th instar larvae of *O. furnacalis*. (**b**,**d**) Relative feeding amount of 5th instar larvae after knockdown of α-amylase or lipase for 3 h. (**e**,**g**) Relative expression levels of α-amylase and lipase at ZT2 in 3rd instar larvae of *D. melanogaster*. (**f**,**h**) Relative feeding amount of 3rd instar larvae at ZT2 of *D. melanogaster*. Bars represent the mean ± SE, * *p* < 0.05, **** *p* < 0.0001. Each treatment was repeated 3 times with 10 individuals per replicate. Different letters above the bars indicate significant differences at *p* < 0.05 as determined by Duncan’s multiple range test.

**Figure 6:**
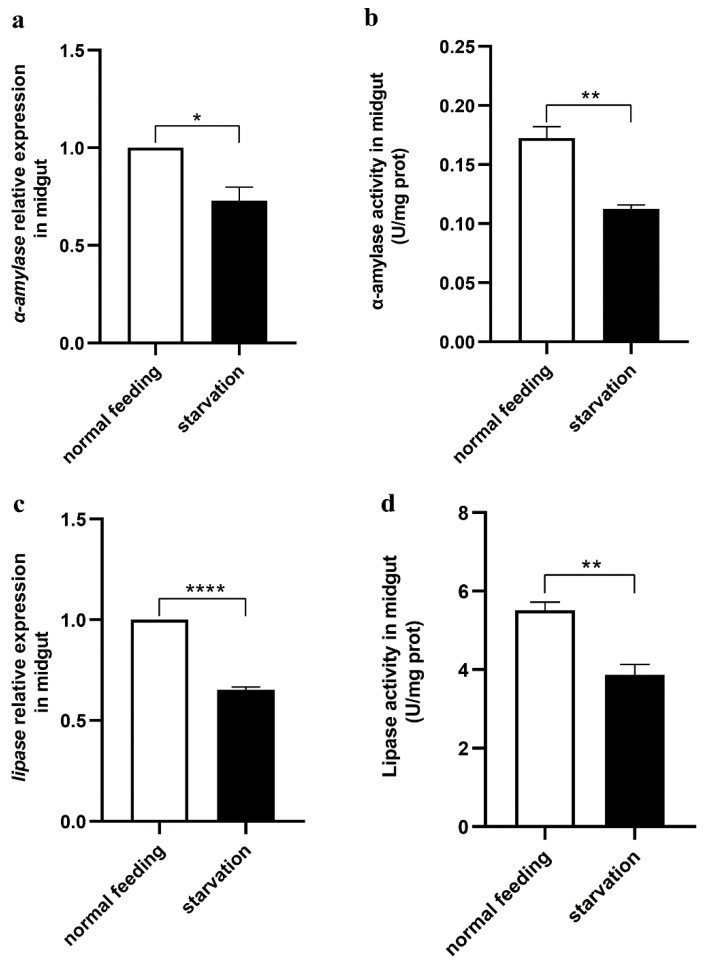
Effects of feeding on midgut α-amylase and lipase. (**a**,**c**) Relative expression levels of α-amylase and lipase in the midgut between larvae with normal feeding and 6 h starvation. (**b**,**d**) The enzyme activities of α-amylase and lipase in the midgut between larvae with normal feeding and 6 h starvation. Each treatment was repeated 3 times with 10 individuals per replicate. Bars represent the mean ± SE, * *p* < 0.05, ** *p* < 0.01, **** *p* < 0.0001.

To clarify the relationship between feeding behaviour and the digestive system in the midgut, we downregulated *α-amylase* and *lipase* and determined the food intake of *O. furnacalis* larvae. The results showed that both *α-amylase* and *lipase* in the midgut were effectively knocked down, and feeding was significantly decreased in the *α-amylase*- or *lipase*-downregulated larvae (Figure 5a–d). Moreover, we also knocked down midgut-specific *α-amylase* and *lipase* through a genetic approach in the UAS/GAL4 transgenic system in *Drosophila*, and similar results were acquired (Figure 5e–h), demonstrating that midgut digestive enzymes affect the feeding of animals. In contrast, larvae in the starvation state had significantly lower expression and activity levels of both α-amylase and lipase (Figure 6). These data indicate that feeding and the secretion of digestive enzymes are interactional.

### 3.5. mTOR Regulates α-Amylase and Lipase Separately via the Transcription Factors c-Myc and PPARγ

**Figure 7:**
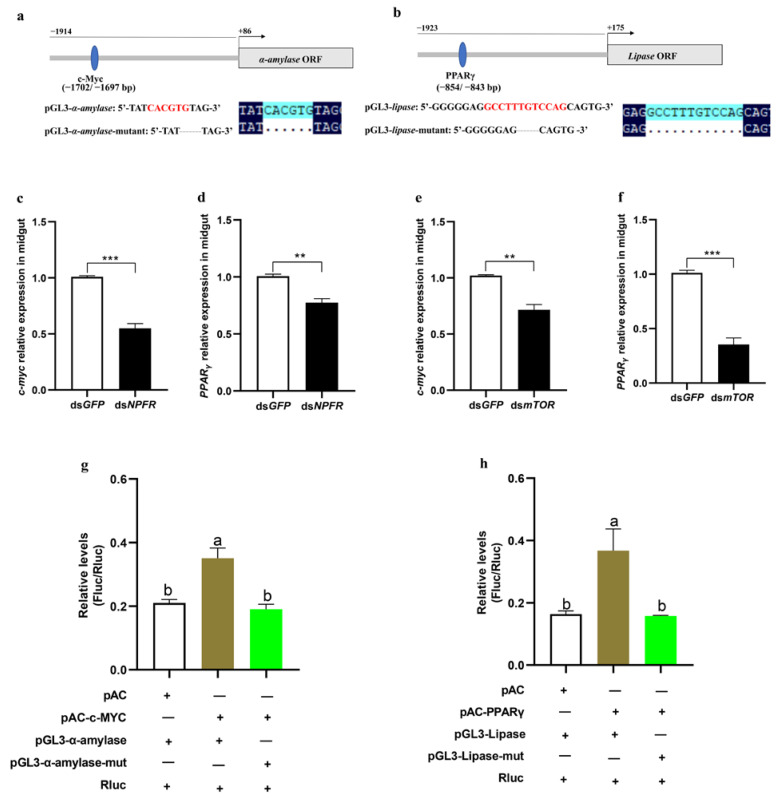
Identification of α-amylase and lipase targets. (**a**,**b**) Schematic representation of the location of *c-Myc* and *PPARγ* response elements in the α-amylase and lipase promoter regions, respectively. Sequences of the predicted *c-Myc* and *PPARγ* response elements. Red letters show the core nucleotides of *c-Myc* and the *PPARγ* binding motif. The grey bar and box indicate the promoter and coding region, respectively. The blue ellipses indicate *c-Myc* and *PPARγ* response elements. Numbers indicate the distance from the translation initiation site of α-amylase and lipase. (**c**–**f**) Relative expression levels of *c-Myc* and *PPARγ* in the midgut after NPFR and mTOR, respectively, were knocked down in 5th instar larvae for 24 h. (**g**) Luciferase reporter assays were performed after cotransfecting pAC and pAC-*c*-*Myc* or pGL-3-α-amylase and pGL-3-α-amylase-mut of the α-amylase binding sites. In all cases, cotransfection with *Renilla reniformis* luciferase was performed. (**h**) Luciferase reporter assays were performed after cotransfecting pAC and pAC-*PPARγ* alone or with pGL-3-lipase or pGL-3-lipase-mut of the lipase binding sites. In all cases, cotransfection with *Renilla reniformis* luciferase was performed. “+” indicates presence, while “–” indicates absence. Each treatment was repeated 3 times. Bars represent the mean ± SE, ** *p* < 0.01, *** *p* < 0.001. Different letters above the bars indicate significant differences at *p* < 0.05 as determined by Duncan’s multiple range test.

To further explore how the insulin pathway impacts *α-amylase* and *lipase*, we first identified a 2000 bp promoter region of both enzymes (Supplementary Figures S4 and S5), predicted their binding sites in the promoter sequence using the PROMO program, and found the potential transcription factors *c-Myc* and *PPARγ* in the promoters of *α-amylase* and *lipase*, respectively (Figure 7a,b). To determine whether *c-Myc* and *PPARγ* can be regulated by the NPF and insulin systems, the expression levels of *c-Myc* and *PPARγ* were measured by knocking down *npfr* and *mtor*, respectively. The results showed that both *c-Myc* and *PPARγ* were dramatically decreased in both the *npfr-* and the *mtor*-downregulated larvae (Figure 7c–f).

To determine whether *c-Myc* and *PPARγ* directly act on the respective promotors of α-amylase and lipase, the promoter sequences of α-amylase (−1914 to +86) and lipase (−1923 to +175) were separately subcloned into the pGL3 vector, and *c-Myc* (amino acids 1-87) and *PPARγ* (amino acids 1-327) were separately subcloned into a pAc5.1b/V5/His vector to obtain the fusion protein of c-Myc or PPARγ integrated with V5/His tag in Sf9 cells. Next, the constructed pGL3-α-amylase (−1914 to +86) and pAc5.1b-*c-Myc* as well as pGL3-lipase (−1923 to +175) and pAc5.1b-*PPARγ* were separately cotransfected into Sf9 cells to perform dual luciferase reporter assays. The empty vector pAc5.1b or mutants of the binding site in the promoter region were used as the control. The results showed that the luciferase activities were significantly increased only when pGL3-α-amylase was bound to pAc-*c-Myc* and pGL3-lipase was bound to pAc-*PPARγ* (Figure 7g,h). These data demonstrate that direct binding to the promoters of α-amylase by *c-Myc* and lipase by *PPARγ* is required for transcriptional activation of these two enzymes.

### 3.6. Feeding and Digestion Are Regulated via Feedback between Gut and Brain NPF

**Figure 8:**
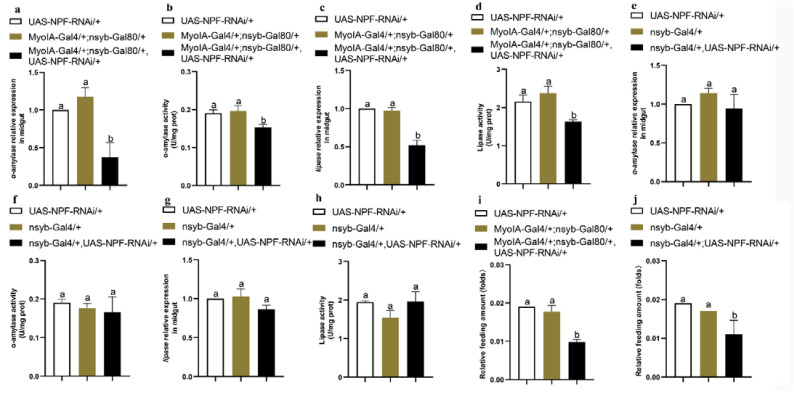
Effects of brain and gut knockdown of *npf* on larval feeding and digestion of *D. melanogaster*. (**a**–**d**) The expression and activity of α-amylase and lipase when npf was knocked down in the gut. (**e**–**h**) The expression and activity of α-amylase and lipase when npf was knocked down in the brain. (**i**) Relative feeding amounts of larvae after specific knockdown of *npf* in the gut. (**j**) Relative feeding amounts of larvae after specific knockdown of *npf* in the brain. Each treatment was repeated 3 times with 10 individuals per replicate. Different letters above the bars indicate significant differences at *p* < 0.05 as determined by Duncan’s multiple range test.

**Figure 9:**
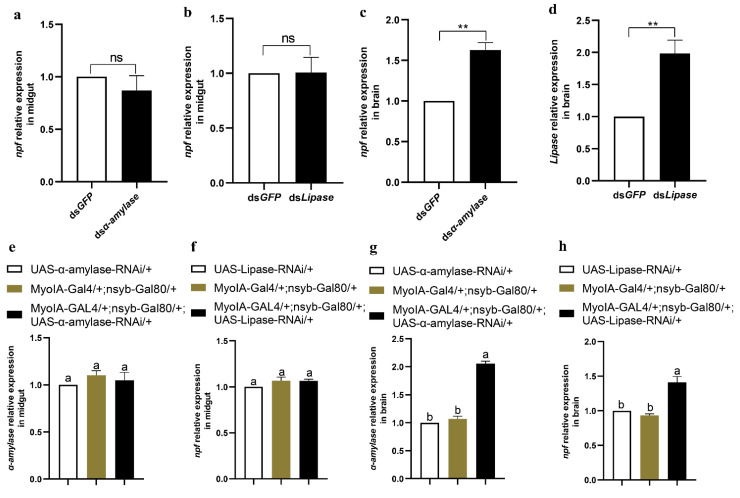
Effects of α-amylase and lipase on *npf* in the midgut and brain of *O. furnacalis* and *D. melanogaster*. (**a**–**d**) Relative expression levels of *npf* in the gut and brain after injection of ds*α-amylase or* ds*lipase* for 3 h in 5th instar larvae of *O. furnacalis*. (**e**–**h**) Relative expression levels of *npf* in the gut and brain at ZT2 in third instar larvae of *D. melanogaster*. Each treatment was repeated 3 times with 10 individuals per replicate. Bars represent the mean ± SE, ** *p* < 0.01. ns indicates no significant difference. Different letters above the bars indicate significant differences at *p* < 0.05 as determined by Duncan’s multiple range test.

Previous studies have shown that *npf* is mainly expressed in the midgut and brain of *O. furnacalis* [7]. To determine which tissue NPF regulates feeding and digestion, we employed *Drosophila* to specifically knock down NPF in the brain and gut, respectively, and investigated larval feeding and the expression and activity of α-amylase and lipase. The results showed that larvae with knocked down NPF in the gut rather than the brain had significantly reduced expressions and activities of α-amylase and lipase (Figure 8a–h), indicating that gut NPF regulates the insulin pathway and thus α-amylase and lipase in the midgut. However, knocking down NPF in either the brain (nsyb-Gal4/+;UAS-NPF-RNAi/+ as treatment, UAS-NPF-RNAi/+ and nsyb-Gal4/+ as controls) or gut (MyolA-Gal4/+;nsyb-Gal80/+,UAS-NPF-RNAi/+ as treatment, UAS-NPF-RNAi/+ and MyolA-Gal4/+;nsyb-Gal80/+ as controls) significantly reduced larval feeding (Figure 8i,j). Thus, we knocked down *α-amylase* and *lipase* in the midguts of both *O. furnacalis* and *D. melanogaster* to determine whether the status of the digestive system may be fed back on brain NFP. Interestingly, the results showed that *npf* expression in the brain rather than the midgut significantly increased after knocking down *α-amylase* and *lipase* in both *O. furnacalis* (Figure 9a–d) and *D. melanogaster* (Figure 9e–h), indicating that the brain NPF, as the controlling centre, is involved in sensing feedback from the midgut digestive system, suggesting that gut NPF may regulate the digestive system, whereas brain NPF may regulate feeding behaviour.

## 4. Discussion

RNA interference (RNAi) has been widely used for studies of gene function in animals [49,50,51,52]. In this study, we constructed pET28a (+)-BL21(DE3) RNase III target genes to efficiently produce a large batch of different ds*RNAs*. Larvae fed artificial diets including ds*RNA* may continuously experience interference with target genes and reduced mechanical damage.

The insulin signalling pathway is highly conserved between insects and mammals [53,54] and regulates a number of physiological functions, such as carbohydrate, lipid, and protein metabolism; tissue growth; and longevity. In *Drosophila*, the NPF system regulates feeding via the InR (insulin receptor)/PI3K/S6K pathway [28], and insulin signalling in mushroom body neurons also regulates feeding behaviour [27]. The mTOR is a hub in the insulin signalling pathway, and its downstream transcription factors, such as HIF1α, c-Myc, FoxO1, SREBPs, PPARγ/PPARα, and TFEB, have been reported to play key roles in regulating the metabolism of glucose and lipid [55]. The mTOR-mediated transcription factors HIF1, c-Myc, and FoxO1 are important for the regulation of cellular glucose metabolism. Among them, overexpression of HIF1 and c-Myc induces an increase in glucose metabolism enzymes and glycolysis levels in the tumour cells [56]. In addition, mTOR is also involved in the transcription factors SREBPs, PPARγ/PPARα and TFEB in lipid metabolism, which is related to the development of tumours, obesity, and fatty liver [57]. In this study, we testify that the transcription factors *c-Myc* and *PPARγ* are direct targets of α-amylase and lipase, respectively.

Feeding and digestion are essential for the maintenance of a normal energy supply, development, and reproduction; homeostasis in food intake and efficient food digestion are important, and the quality and quantity of foods determine the secretion of digestive enzymes [58,59]. In this study, we clarify that the digestive enzymes α-amylase and lipase in the midgut are closely related to larval feeding and are regulated by the NPF/NPFR system via the insulin signalling pathway. Insect α-amylase and lipase mainly decompose the respective starch and lipids in foods into reducing sugars and fatty acids [60,61]. When they are inhibited, insect nutrition is impaired, which leads to retarded growth and development and even death [37].

In this study, knocking down NPF with ds*NPF* in *O. furnacalis* significantly decrease the expressions of α-amylase and lipase. To further determine which tissues of NPF regulates their expressions, we used *Drosophila* to tissue-specifically knock down NPF in the brain and gut, respectively, and detect their expression in the midgut. This cannot be conducted in *O. furnacalis* due to it lacking as convenient and mature of a transgenic system of the UAS/GLA4 as *Drosophila*. *Drosophila*, as a model animal not only used in insects but also in Mammalia for studies of mechanisms, is a useful genetic tool to conveniently work on target genes of tissue-specific cells.

**Figure 10:**
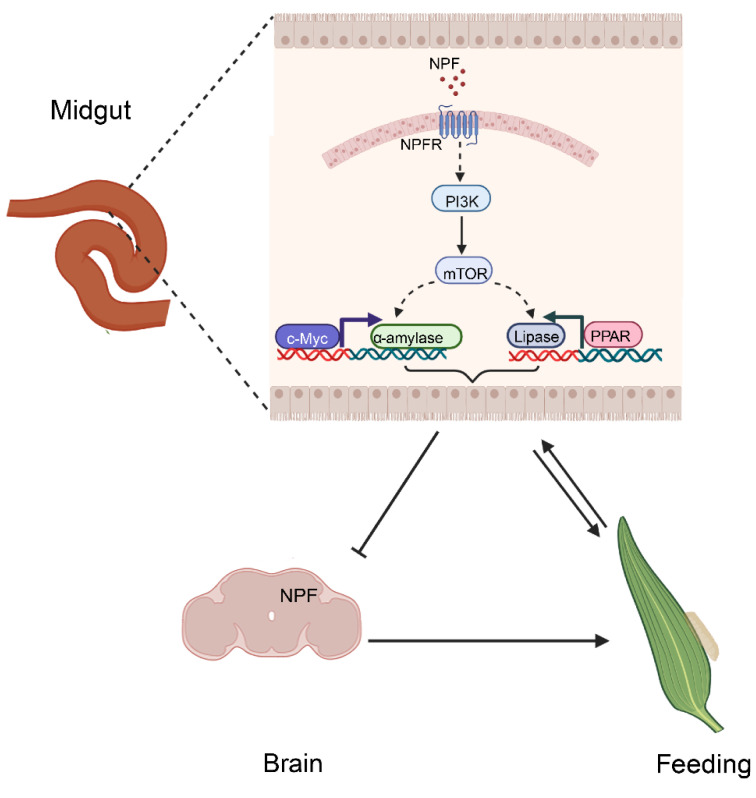
Schematic representation of the NPF signalling pathway cascade in feeding and digestion in the midgut of *O. furnacalis*.

In *Drosophila*, activation of NPF signalling in the brain promotes adult feeding [62], and *Drosophila* with NPF knockdown results in decreases in food amount, whereas overexpression of NPF results in the opposite phenotype [2]. Here, we found in *Drosophila* that gut NPF rather than brain NPF regulates α-amylase and lipase via *c-Myc* and *PPARγ*, respectively. Importantly, the status of α-amylase and lipase may negatively feed back on brain NPF, further testifying that the brain NPF is also important for regulation of feeding through other paths and is the regulatory centre through which insect feeding is controlled—the complex mechanism of feeding regulation. For example, brain NPF rather than gut NPF regulates feeding through the juvenile hormone (JH) pathway [24]. Here, a regulatory cycle of feeding behaviour and digestion in the midgut of *O. furnacalis* larvae is shown in Figure 10.

## Data Availability

The datasets generated during and/or analysed during the current study are available from the corresponding author on reasonable request.

## Supplementary Materials

The following supporting information can be downloaded at: https://www.mdpi.com/article/10.3390/cells12010194/s1, Figure S1: Schematic diagram of the ds*RNA* production system; Figure S2: Effects of RNA interruption; Figure S3: The relationship between NPF and PDK/AKT/PKC in insulin pathway in midgut of *O. furnacalis*; Figure S4: The nucleotide promoter sequences of α-amylase; Figure S5: The nucleotide promoter sequences of lipase; Table S1: Primer sequences of synthesis dsRNA used for larval treatment *via* diet-feeding; Table S2: Primer sequences of synthesis dsRNA used for larval treatment *via* injection; Table S3: Primer sequences for the qPCR assay; Table S4: Primer sequences for dual luciferase reporter assay.
