# Supplementary material for: Synergism of Feeding and Digestion Regulated by the Neuropeptide F System in Ostrinia furnacalis Larvae
Source: Cells. 2023 Jan 3;12(1):194. doi: 10.3390/cells12010194 (PMC9818795; doi:10.3390/cells12010194)
Supplement: Supplementary file 1 [file cells-12-00194-s001.zip › cells-2093930-supplementary.pdf]

Supporting Information

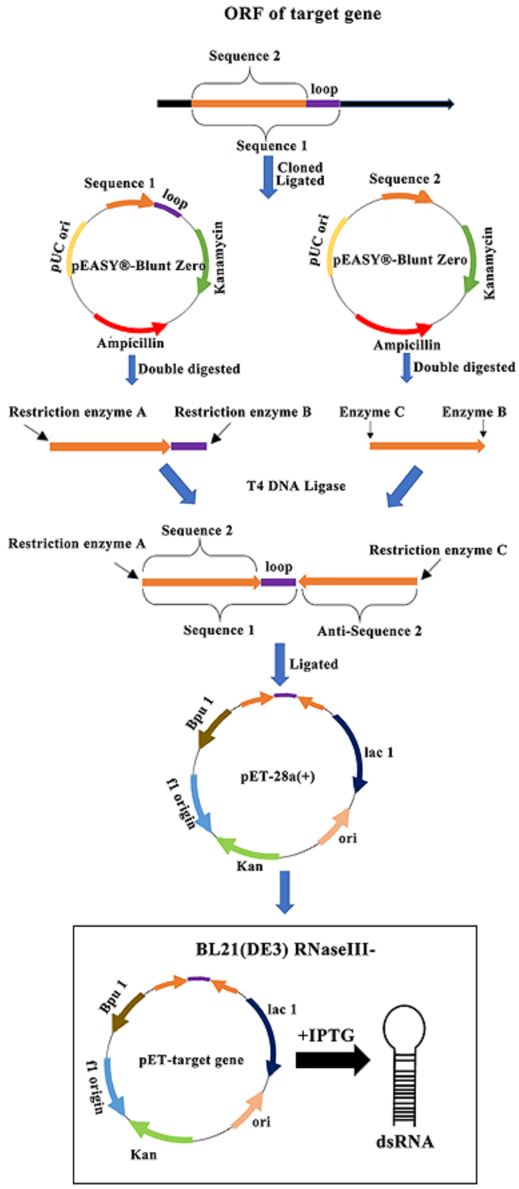

Figure S1. Schematic diagram of the ds*RNA* production system.

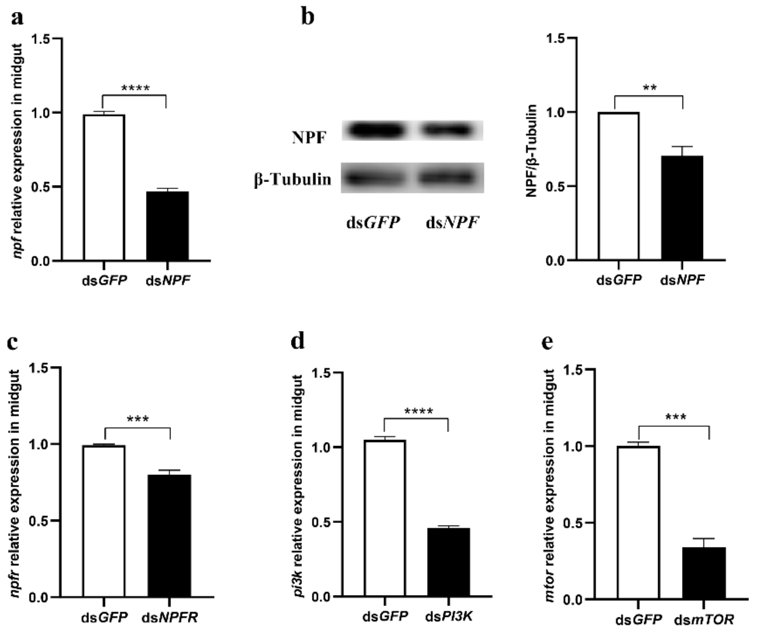

Figure S2. RNAi. (**a**) the relative expression levels of *npf* in midgut of the 5th instar larvae after larvae were treated with ds*NPF* for 24 h. (**b**) The protein level of NPF was analyzed by Western blot and the gray scale value of the protein strip was analyzed by Image J software. (**c-e**) the relative expression levels of *npfr*, *pi3k* and *mtor* in midgut of the 5th instar larvae after larvae were treated with ds*NPFR*, ds*PI3K* and ds*mTOR* for 24 h, respectively. Each treatment was repeated 3 times with 10 individuals per replicate. Bars represent the mean ± SE, ** $p < 0.01$, *** $p < 0.001$, ****$p < 0.0001$.

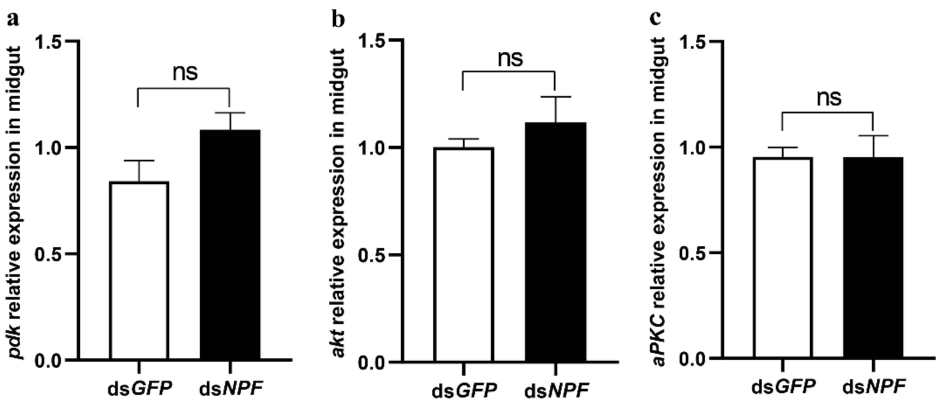

Figure S3. The relationship between NPF and PDK/AKT/PKC in insulin pathway in midgut of *O. furnacalis*. (**a-c**) Relative expression levels of *pdk, akt* and *pkc* in insulin signaling pathway after the 5th instar larvae were knocked down NPF for 24 h, respectively. Each treatment was repeated 3 times with 10 individuals per replicate. Bars represent the means ± SE. ns indicates no significant difference.

>*α-amylase* -promoter (-1914 to +86)

```
GAAAGATATTTCAAAAATTTATAATAATATGATCTTTGATATTAATGCAAATAATGCAATAAGTAATCAACAGCAT
TATTTTTATTACCTACATTAAATCAAAGGTCATTTAGGTCACACATGTGTGATTTGATTGTATTATTTTCAATTGA
TACCCTGTGTTTTAGGGCTCCGCAAAACTTTTGAATAAACGGAACTGTTGCAAAATCTATCACGTGTAGCTTTACG
TACACTTTTCAGTCTATAATTAGCCATTTTTTTTTCAGAAAACTTTCAATTGGTGTATTTTTGTTATTAACAACAT
GAAAAAAAAATATTTTAAACCAAAATATTACATCCGAAATCACTGAACAATAATTTAAAAAAAATAGGAACATAAC
AGTTTTACAGAAAAATATACTGAAACTCTTATTATAATTTTTAGCATTTTGTGTATTAATTAAGGTACCTAATATT
AATTAATACAAAAAAATTGCTGTTCTTGATACACGTAAAAGTAAGTATTACATTTATCATGCAAGAACCCTCTTCA
AGCGAGTAAAAATCACACTTGACCGGTATAAGACCGGATAGGTGTAATTATCAGTTGATTAAATGAGATCGATATC
ACTTATACCTTCACCGAGGGACTGATAATATTTCAGCAAGCAATCAGATTGGATAACAATGCATTTTTGATTGTTG
ATGTAAGCTTCCCCCCTAGACAAGACAACCGTGTTGCAGTTTCATTGCGATATCGCTTCGTTCCGTGCCCGTGTAG
CGATCGAAATTTCATCTTTCAAGAATGTTTCCATGCAAGGTTTCTGCGGCAAAGACTACATTTTTCAAAAATGATA
TGCTTGTAATAAAACTAAATGGTGGTACCATTATAGCATTGACCCTGGACTTAACCTGATCTTTATCCTGCCCTGA
AACACCTAGATTACTTCTTCAAAGATATACTAAGCAAGCAAGTATCAGTATGTACGATCAATTTTGAATTTTACAG
TTCGAAAGTTTTTAAATAACTTGATCGTTAGGATTATCCCACAACTGTTGAATAGCTTCCCACTAATGCCTCGAAT
AGTAATAAAAAGTCAGAATCTCAGGTAGTGCAAGTAGATCAATGAAACTAGCAGCCATTGCGTGAGGGTTCAAATA
AAAATACTTATTCCTTAACAACAAGAGGGTTAATTGTTACAATAAAACCTTTCCCACGCCACTGAATGTTCCGTTT
AAACATCAATTAATCGTTTTAATTCAAAAGCATGAATTTATATCGCTAATTAAGCTCATATTGCGTACCATTAATT
CTTACCTATCCTGATGACCGTAATTAAATTCATATTTCGGGTGTGAAGAATGATTAACTTTTAATTACTTGTATTG
TTCAATATAATAATTAGTATCATCAATAATATTACGCGTAACCCAATTGCGGATGAATAACAACTAACTGGTATAC
AAATAAACCACGATCGAGAACATTCGAACTGCTTCTTTTCTACAAAGTTTTCTTTAGAAAAGTTTTCACAAACGAT
TATATGAATGTAATATTGTGACTTGAAACTTTCTTCTAACTGAGAAACCCGACCATAGTCTTTTAAGAGAGTGTCT
TTGTCAGAGAAAAAGAAAAAGGGTGTTTTTTTATCCTGCCATCTGAAAAAGAATTGCTGTTTTGTCTTATGGATGA
ATGTTATTTCAACATAAGTGTTCAAACTGTATTTCTAAAATGAGAAGGGAAAGCATCCTGGAAAATGCTTGATTAA
TAAATTGAAAGATTCCGATTTCCTTTTTATAGAACACTAAGGATGGAATATTGCTTTAGATAGGTATTTTTACGGA
GAATCTCAGCAGATTGGAATCAAAAATATTTCAACTAGTCATACAAAGATAAAATTCAGTCGATATAAATACACTA
CCACCCAGTCTTTTATCATTAGCACCGCCAGGGCGATCGCGATAGTGTATCTGTATCGGAGCATAATTGGGCAATT
CGCGAGCCGAGGTGGTCAGTAACC
```

Figure S4. The nucleotide promoter sequences of α-amylase. The α-amylase promoter sequences were cloned to the pGL3 vector to conduct the dual luciferase reporter assay. The binding sites of transcription factors *c-Myc* is underlined.

>*Lipase* -promoter (-1923 to +175)

CATCGTAAAGCAGGGAAGCGCTGGACGCAGGCTGCTACCAATCGACCAACATGGAATGCATTGGGGGAGGCTTATG

TTCAGCAGTGTACGTCCTATGAAATGATGATGATGAACCGTCGGCCGACATAAAGTCTGTAGTCTGCGGCTAGGCT

AAAAAGGAAACGATGAGGCATGAGCTACATTCGACATGAAAAGCAGATATGCATAAGTTACGGGTGGGTTCACCAC

AATAGTCCTGTGGCGAAGGGATTCGAACATGCGTTCTTGGGATCTCGAGTCGGCCAATCCGACACCGACACCATTG

GGGTATTGTTGTATTTTAGAATACGGTCCACAGTTATGTTTATTTATTGACTTTATTAGGATTTATTAACCAGGTA

TCTTCTATCTATATCTATAAAAGCGAAAAGTCACTAATTGACTGACACACTCATCACGAAATCTCAGAAACTACAA

GTGCTAGGAGTCTCAAATATTGCATGGGGGTTCACTAAGAACGGATTTTACGAAACTCCACCCTTAAGGGGGTAAA

ACGGGATCCACGCGTACGAAGTCGCGAAGCTTTACACCAATATGTTTTCGCATACAATACATAACTGAGAAAATCA

TTTAACCGTAGTCAACTAGTGTGCCTTGATGTGCTGTCAGTCGTCAGTTTTTAGTCTCTTATCAAACCATTTACCA

TCATCTAACGTATGAATTTGTGGTGAGCTTACAATAATGATGTATGTACGCCTATTCTGTCTATTATCTATGAAAT

CTCGGCTTGACATCGAGCCGCTCACAAGATCCTAACCAAATTCAAAGATTGACAGATGTTCAGCCCAGTCGAACAT

ACATACCTAATATCGAGACCCTTATCGCTGGAACTGACAGACGACGCCTCTGGGACTCGAATACACGACAAACCTC

GCACTGTGCATGGGCTGTGACGTGTAATAGAAGCATCAATAAAGTTTTACAAGTTACTGGCTTTAGGCTAAATCCT

TCATTTGTCTTTGGTAAATTAGAAAGGAAGGTGCCTGGATGCGGGTGGCGCAGGACCGGTCTTTGAAAAAAACCTT

GGGGG**AGGCCTTTGTCC**AGCAGTGGACGTCTTTCGGCTGAAACGAACGAACGAAATCAGAAAGGGTCGTGCTCCTG

TAGTAAATACTAAATGACTTGGTAATGATGATGATGAATATGTACCAGTGCAGTAAATTTACAAACTCGTTTTTAA

AGGTTTACAATAGTATATTTATTATTTTGTAGATACCCGCTAGGCAATTAACTAATCTTCATCATCGTCATTTCAG

AAACAGGACGTCCACTTCTGAACATAGGCCTCCCCCAACACAAATAGGTAGGTAGGTCTAATGAAAATAAATAACT

TAGGTAGGGTTCCAAGACGTGGTCTGCACTCGAGCTTAAGATTATGACATTAAAGGTTATTTTAATTTTCAACTTC

ACAAAAAAACAATTGTCATTTTCGCACTGATTTTCAAGATAAGGGGGTGGCCCTTATTTTTTGCCAAAGCATAATT

TTAGCTATTAGATAAAACCAAATCAATTTGATCATATTATAGTAGGTACACTTTGAGATAGTTAAAATGTGATTCC

ACAAAAAACAATTGTCATTTTCGCACTGATTTTCAAGATAAGGGGGTGGCCCTTATTTTTTGCCAAAGCATAATTT

TAGCTATTAGATAAAACCAAATCAATTTGATCATATAGTACACTTTGAGATAATTAAAATGTGATTCAACAAAAAA

CAATTGTCATTTTCGCACTGATTTTCAAGATAAGGGGGTGGCCCTTATTTTTTGTCAAAACATAATTTTAGCTATT

AGATAAAACCAATTCAATTTGATCATATTATACATAGTACCTACACTTTGAGATAATTAAAATGTGATTCCACAAA

AAAAAACAATTGTCATTTTCGCACTGATTTTCAAGATAAGGGGGTGACCCTTATTTAGCCAAAACATAATTTTAAC

TATTAGATAAAACCAAATCAATTTGATCATATAGTACACTTTGAGATAATAAAAATGTGATTATTATCTACGCATT

GATTATCTAAATTGATATGTTTTATAAATAAGCATTTTATTCTCGA

Figure S5. The nucleotide promoter sequences of lipase. The lipase promoter sequences were cloned to the pGL3 vector to conduct the dual luciferase reporter assay. The binding sites of transcription factors $PPAR\gamma$ is italicized.

Table S1. Primer sequences of synthesis dsRNA used for larval treatment *via* diet-feeding

Primer name		Primer sequence (5’-3’)	Restriction Site
ds*NPFR*	Sequence 1-F	CCGGAATTCAGGCATACAGGACCCAAACG	EcoRI
	Sequence 1-R	TGCTCTAGACTTGATGGGCCAGTCTTCTAT	XbaI
	Sequence 2-F	CCGCTCGAGAGGCATACAGGACCCAAACG	XhoI
	Sequence 2-R	TGCTCTAGAAGGGGAGGCGAGGGTGAAG	XbaI
ds*PI3K*	Sequence 1-F	CCGGAATTCGTCAGTCAAGGTCCCAGGTG	EcoRI
	Sequence 1-R	TGCTCTAGACTTGGGAGTCAGTCAGAAAC	XbaI
	Sequence 2-F	CCGCTCGAGGTCAGTCAAGGTCCCAGGTG	XhoI
	Sequence 2-R	TGCTCTAGATGGAACTGTATGAGGGGGTA	XbaI
ds*mTOR*	Sequence 1-F	CCGGAATTCTGCCTCCTTATCTTGCCGTA	EcoRI
	Sequence 1-R	TGCTCTAGATGCAGGTCGTACATCGACAG	XbaI
	Sequence 2-F	CCCAAGCTTTGCCTCCTTATCTTGCCGTA	HindIII
	Sequence 2-R	TGCTCTAGACCGTTGTAGTGGAATAGCAC	XbaI
ds*GFP*	Sequence 1-F	TGCTCTAGACACAAGTTCAGCGTGTCC	XbaI
	Sequence 1-R	TATAAGCTTGATATGGCTAACCTGGTTC ACCTTGATGCCGTT	HindIII
	Sequence 2-F	TATCTCGAGCACAAGTTCAGCGTGTCC	XhoI
	Sequence 2-R	ATCAAGCTTACCTGCCAAAACGAACG TTGTGGCTGTTGTAGT	HindIII

Notes: The underline is restriction site.

Table S2. Primer sequences of synthesis dsRNA used for larval treatment *via* injection

Primer name	Primer sequence (5’-3’)	usage
α-amylase-CDS-F	ATGACGACTTTGAAGGCAGT	Synthesis double-stranded RNA for injection
α-amylase-CDS-R	TTACGATACTGGTGCAGTTT	
Lipase-CDS-F	ATGTTTTATTCCGTGTTG	
Lipase-CDS-R	TTAGACAAATGGGTAATATCT	
ds*α-amylase*-F	TAATACGACTCACTATAGGGTGGCTGGCGACGAGAAATA	
ds*α-amylase*-R	TAATACGACTCACTATAGGACTCCCTCCACTCGTACACT	
ds*Lipase*-F	TAATACGACTCACTATAGGCCTTTCGGTAACCTCCATCT	
ds*Lipase*-R	TAATACGACTCACTATAGGGATAAATGCCGTTCCCTCGT	
ds*GFP*-F	TAATACGACTCACTATAGGGAGACAGCGTGTCCGGCGAGG	
ds*GFP*-R	TAATACGACTCACTATAGGGAGAGTTCACCTTGATGCCGT	

Notes: The underline is T7 promoter sequences

Table S3. Primer sequences for the qPCR assay

Primer name	Primer sequence (5’-3’)	usage
q*NPF*-F	CCGCATTTACTCCTACCACA	Real-time quantitative
q*NPF*-R	CCAACCAAGCGGGTAAGT	
q*NPFR*-F	ATAGAAGACTGGCCCATCAAGAATG	
q*NPFR*-R	CTCGTGGTCCTTCTTCTCCC	
q*PI3K* -F	CTCCAGCCCTAATACCGACTC	
q*PI3K*-R	TGGGGTCCCTTTCAGCGGTTG	
q*mTOR*-F	GCGAAAGGTCACAAATACTT	
q*mTOR* -R	GGCAGATTGAGATCATGGAT	
q*PDK* -F	GACTGGCGAACATAATGAAAG	
q*PDK*-R	CGCAGAACTGACTCAACACCG	
q*AKT* -F	TCGAGTTCGTGAAGGTGCTG	
q*AKT* -R	GATGCTTGGTCTTCTTGAGC	

q*aPKC* -F	GATCACGAGGGGCACATCAA	PCR (qPCR)
qa*PKC* -R	GTCCACGCTGAAGCCATACT	
q*α-amylase* -F	TCGTGGATGCTGGTGTAGAC	
q*α-amylase* -R	ATTTTGCTTCAGTGCTGGCG	
q*Lipase* -F	CCAACCGTGATTCTCGTCCA	
q*Lipase* -R	ATTCATGATAATAAAACTGTATAGG	
q*c-Myc* -F	AGATTCCAAGAGAGAAGAGT	
q*c-Myc* -R	CTCATCGTCACCACCATCTG	
qPPARγ-F	ACACCACCGCCAAAAGTATG	
qPPARγ-R	AACCCACCAAACCAGCGTAA	
actin-F	ACGGAGGTGGTAACCATCAACA	
actin-R	ACGCCTCCTTCTTGGTGTCG	
Dm-q*NPF*-F	CATCCTGGTTGCCTGTG	
Dm-q*NPF*-R	TGTTGACATCGTTCTTTCG	
Dm-q*α-amylase* -F	ACCAACCCATCTCCTACAAGC	
Dm-q*α-amylase* -R	CTCTTGCTGCTGGGGCTGG	
Dm-q*Lipase* -F	CTATTTCTGATTGCGGTGAG	
Dm-q*Lipase* -R	AGGAAGAACTCAGCATGCCG	
Dm-actin-F	CAGAGCAAGCGTGGTATCCT	
Dm-actin-R	CTCATTGTAGAAGGTGTGGTGC	

Notes: Dm represents *Drosophila melanogaster*

Table S4. Primer sequences for dual luciferase reporter assay

Primer name	Primer sequence (5’-3’)	Restriction Site
α-amylase-PGL3-F	CGAGCTCGAAAGATATTTCAAAAATTTA	SacI
α-amylase-PGL3-R	TCCCCCGGGGGTTACTGACCACCTCGGCT	SmaI
Lipase-PGL3-F	CGAGCTCCATCGTAAAGCAGGGAAGCG	SacI
Lipase-PGL3-R	TCCCCCGGGTCGAGAATAAAATGCTTATTA	SmaI
α-amylase-mutant-F	TAGCTTTACGTACACTTTTCA	
α-amylase-mutant-R	ATAGATTTTGCAACAGTTCCG	
Lipase- mutant -F	AGCAGTGGACGTCTTTCGGCT	
Lipase- mutant -F	CCCCCAAGGTTTTTTTCAAAGA	
c-Myc-CDS -F	CAGTGTGGTGGAATTATGTCGTCGTACAAA CCTATAGATTCCAAG	

c-Myc-CDS -R	GGCCCATAATAAGCTTTATCCTTCCGTTGG GGCTGC
PPARγ-CDS -F	CAGTGTGGTGGAATTATGGGTATACAAGAT TTAC
PPARγ-CDS -R	GGCCCATAATAAGCTTTATCTGGCTTTTGG TTCTTTTTGT

Notes: The underline is restriction site and labeled green is the homologous recombination sequence
